# Supplementary material for: Basement membrane damage by ROS- and JNK-mediated Mmp2 activation drives macrophage recruitment to overgrown tissue
Source: Nat Commun. 2020 Jul 20;11:3631. doi: 10.1038/s41467-020-17399-8 (PMC7371875; doi:10.1038/s41467-020-17399-8)
Supplement: Supplementary file 1 — Supplementary Information [file 41467_2020_17399_MOESM1_ESM.pdf]

Supplementary Information

Basement Membrane damage by ROS- and JNK-mediated Mmp2 activation drives macrophage recruitment to overgrown tissue

Neha Diwanji and Andreas Bergmann*

University of Massachusetts Medical School

Department of Molecular, Cell and Cancer Biology

364 Plantation Street, LRB 419

Worcester, MA 01605

USA

*Author for correspondence

andreas.bergmann@umassmed.edu

Supplementary Table 1: Frequency of BM damage observed for controls and undead discs

	Genotype	Total number of eye discs	Number of discs with uniform BM	Number of discs with disrupted BM
**Perlecan**	*ey-Gal4*	34	34	0
	ey>p35	37	37	0
	ey>hid,p35	50	0	50
**Laminin**	*ey-Gal4*	18	18	0
	ey>p35	13	13	0
	ey>hid,p35	36	0	36
**SEM**	*ey-Gal4*	18	18	0
	ey>p35	12	12	0
	ey>hid,p35	20	0	20
**TEM**	*ey-Gal4*	3	3	0
	ey>p35	3	3	0
	ey>hid,p35	3	0	3

100% of all undead *ey>hid,p35* eye discs show some degree of damage to the BM as assayed by immunolabeling with Perlecan and Laminin, as well as SEM and TEM, while none of the control eye discs have a damaged BM.

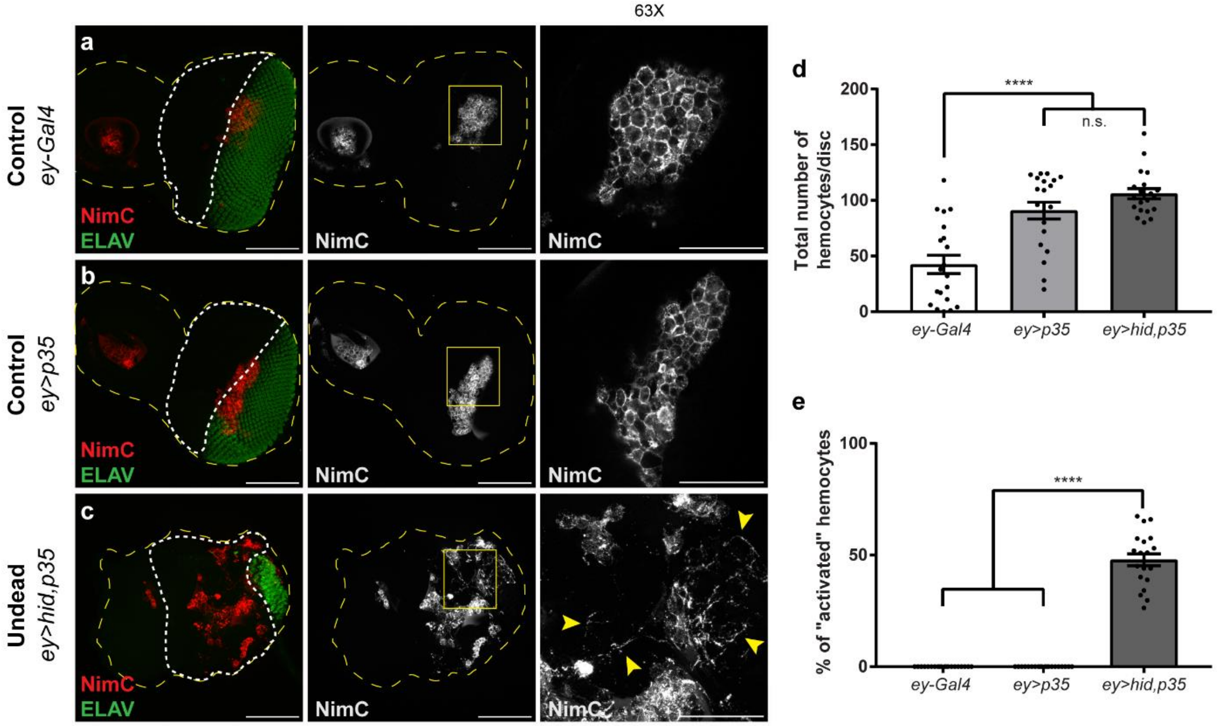

Supplementary Figure 1. Undead discs have increased numbers of hemocytes

(**a-c**) Hemocytes (labeled by NimC; red left; grey middle and right) in control *ey-Gal4* (a) and *ey>p35* (b) discs are present in tight cellular clusters (yellow box magnified at right) at the boundary of the differentiating photoreceptor neurons (labeled by ELAV; green left). In undead *ey>hid,p35* overgrown discs, hemocytes are present as single cells or in small clusters, are spread out over the undead portion of the eye disc and are increased in numbers (c, d). These hemocytes extend cellular protrusions (yellow arrowheads; right) indicating an activated phenotype. Discs are outlined by yellow lines; the *ey-Gal4* expression area is outlined by white dotted lines in (a, b, c). Scale bars, 100µm (left and middle) and 50µm (right).

(**d-e**) Quantification of hemocyte recruitment (d) and activation (e) in control and undead *ey>hid,p35* discs. Hemocyte activation was measured by the percentage of total hemocytes that had one or more cellular protrusions. 48% of total recruited hemocytes in *ey>hid,p35* discs had an activated morphology, while the controls did not show any activated hemocyte (e). Total number and percentage of activated hemocytes were analyzed by one-way ANOVA with Tukey's multiple comparisons test. ****$p < 0.0001$, n.s. = no statistical significance. Data from n=20 discs per genotype analyzed from 4 independent experiments. Source data are provided as a Source Data file.

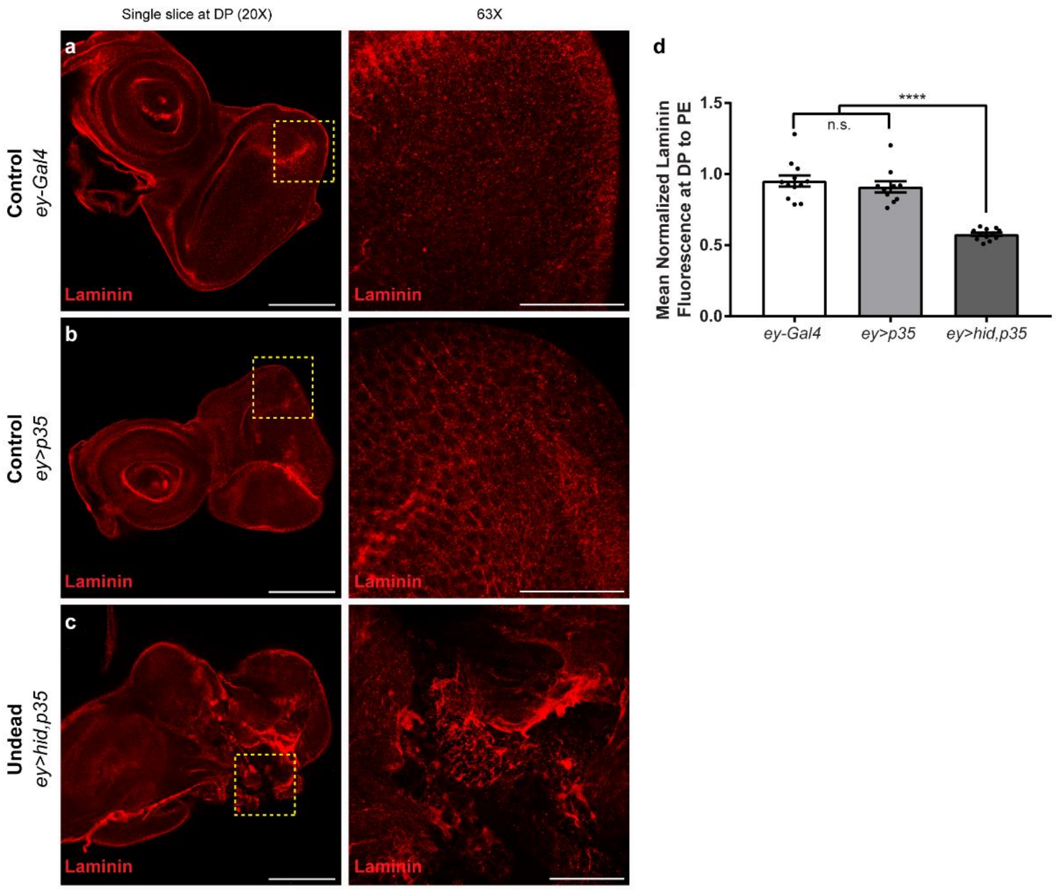

Supplementary Figure 2. Laminin labeling in undead discs

(**a-c**) Representative examples of control (*ey-Gal4* (a) and *ey>p35* (b)) and experimental (undead) (*ey>hid,p35* (c)) eye imaginal discs labeled for the BM with Laminin antibody (red). Single slices (left), yellow squares magnified (right), focusing on the basal side of the DP. Laminin labeling in control eye discs is continuous indicating that the BM uniformly surrounds the discs (a-b). Laminin labeling of undead discs shows areas where the BM is discontinuous or absent with gaps and punctate-like appearance indicating damage (c). Scale bars, 100µm (left) and 50µm (right).

(**d**) Quantification of Laminin labeling reveals that the BM in undead *ey>hid,p35* discs is damaged. Fluorescence intensity at PE was used as internal control for normalization. Quantification of Laminin fluorescence is taken from single slices at the basal surface of DP and PE and represented as Mean Fluorescence ± SEM analyzed by one-way ANOVA with Holm-Sidak test for multiple comparisons. ****$p < 0.0001$, n.s. = no statistical significance. n=12 (*ey-Gal4*), 10 (*ey>p35*) and 12 (*ey>hid,p35*) discs analyzed from 3 independent experiments. Source data are provided as a Source Data file.

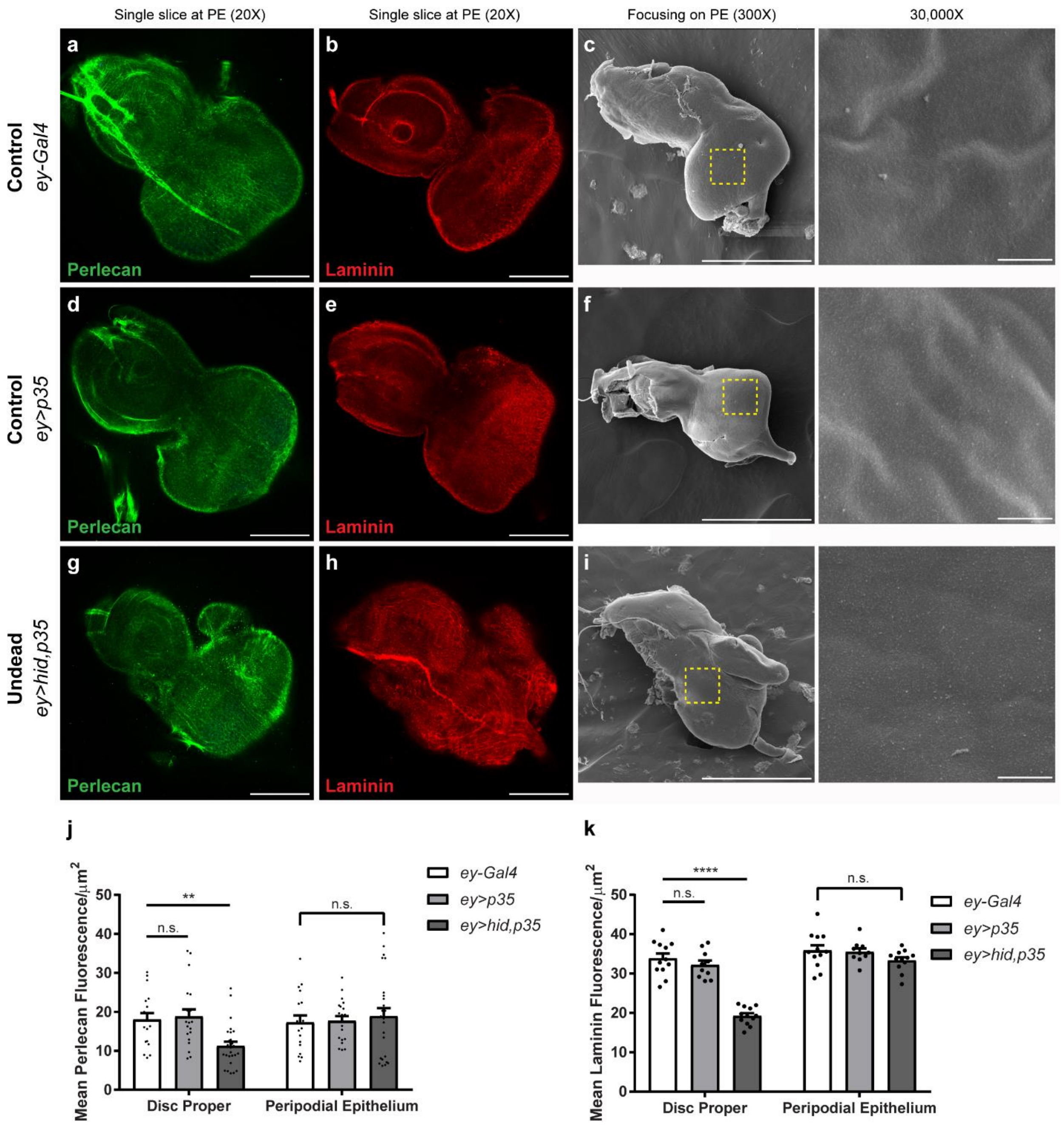

Supplementary Figure 3. The BM on the PE side of undead discs is not damaged

(**a-i**) Representative examples of control (*ey-Gal4* (a-c) and *ey>p35* (d-f)) and experimental (undead) (*ey>hid,p35* (g-i)) eye imaginal discs labeled for the BM with anti-Perlecan antibody (green in a, d, g), anti-Laminin antibody (red in b, e, h) and Scanning Electron Microscopy (SEM) images (c, f, i) focusing on the PE (peripodial epithelium) side. Panels (a, b, d, e, g, h) are single slices focused on the basal side of PE. The yellow square in (c, f, i) is magnified at (right) focusing

on the basal side of PE. Undead discs show similar uniform BM as seen for control discs at the PE side indicating that BM is not damaged. Scale bars, 100µm (a, b, d, e, g, h), 200µm (c, f, i; left) and 1µm (c, f, i; right).

(**j, k**) Quantification of Perlecan (j) and Laminin (k) labeling reveals that the damage of the BM in undead *ey>hid,p35* discs is selectively observed at the basal side of the DP while the labeling at the PE side is comparable among the controls and experimental discs. Quantification of Perlecan and Laminin fluorescence is taken from single slices at the basal surface of DP and PE, and represented as Mean Fluorescence ± SEM analyzed by one-way ANOVA with Holm-Sidak test for multiple comparisons. **p=0.0031, ****$p < 0.0001$, n.s. = no statistical significance

(j): n=18 (*ey-Gal4*), 20 (*ey>p35*) and 26 (*ey>hid,p35*) discs analyzed from 5 independent experiments;
(k): n=12 (*ey-Gal4*), 10 (*ey>p35*) and 12 (*ey>hid,p35*) discs analyzed from 3 independent experiments.
Source data are provided as a Source Data file.

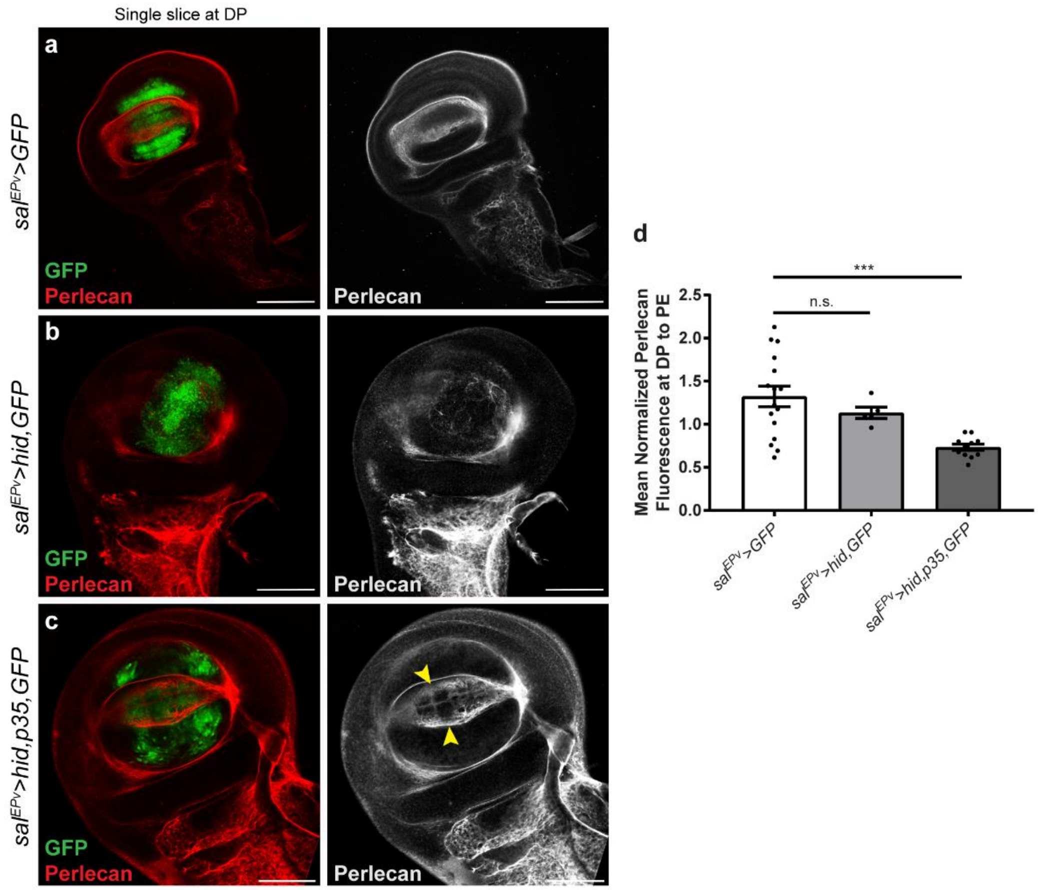

Supplementary Figure 4. Induction of Apoptosis does not damage the BM

(**a-c**) BM detected by Perlecan (red left; grey right). GFP marks the domain where Gal4 is expressed. Apoptosis induced by expression of *hid* does not damage the BM (b). Perlecan labeling is slightly disrupted around the *hid*-expressing area (b; right), but no gaps or absence of Perlecan labeling is observed and is comparable to the GFP-only control (a). Undead tissue created by expression of *hid* and *p35* (c) shows damage to the BM by Perlecan labeling with gaps appearing in and around the *hid,p35*-expressing area indicated by yellow arrowheads (c; right). Panels are single slices focused on the basal side of DP. Scale bars, 100μm.

(**d**) Quantification of Perlecan intensity in taken from single slices at the basal surface of DP and PE. Fluorescence intensity at PE was used as internal control for normalization. Data represented as Mean Fluorescence ± SEM analyzed by one-way ANOVA with Holm-Sidak test for multiple comparisons. ***p=0.0006, n.s. = no statistical significance. Data from n=16 (*sal^{EPv}>GFP*), 5 (*sal^{EPv}>hid, GFP*), and 11 (*sal^{EPv}>hid,p35,GFP*) discs analyzed from 3 independent experiments. Source data are provided as a Source Data file.

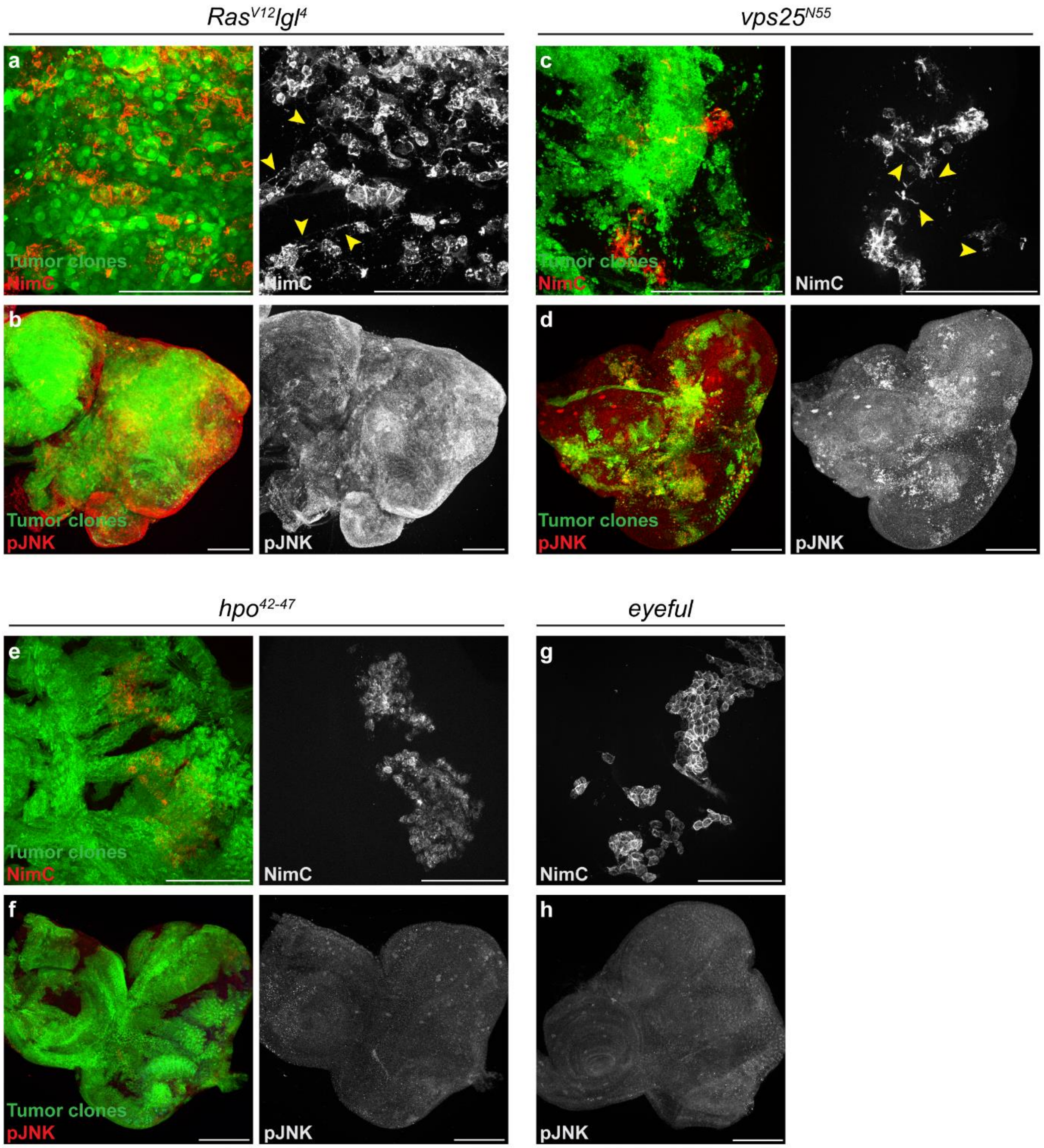

Supplementary Figure 5. Hemocytes and JNK activation in different tumor models

Except for *eyeful* (g,h), tumor clones were generated by the MARCM technique using *ey-FLP* in the eye imaginal discs and are marked by GFP (a-f). *eyeful* is uniformly expressed in the *ey-Gal4* domain (exact genotype: *ey>Dl,lola,psq*). Scale bars, 100μm.

(**a, c, e, g**) Hemocytes are detected by NimC (red left; grey right). Hemocytes attached to mosaic discs of neoplastic tumor models such as *Ras*^{V12}*lgl*^{4} (a) and *vps25* (c) show an activated morphology, around tumor clones as indicated by yellow arrowheads (right). In contrast, although the hyperplastic tumor models such as *hippo* mosaics (e) and *eyeful* (g) have a high number of hemocytes attached to the discs, these hemocytes do not have an activated morphology. The hemocytes present are tightly clustered, are not present as single cells, and do not have extended cellular protrusions (right). Quantified in Supplementary Figure 6c, d.

(**b, d, f, h**) Activation of JNK is detected by phospho-JNK (pJNK) antibody (red left; grey right). The neoplastic tumor mosaics show upregulated active JNK (b; d), while the hyperplastic models do not have activated JNK (f; h), suggesting a correlation between hemocyte activation, JNK activation with production of ROS and damage to BM (Figure 2). Quantified in Supplementary Figure 6e.

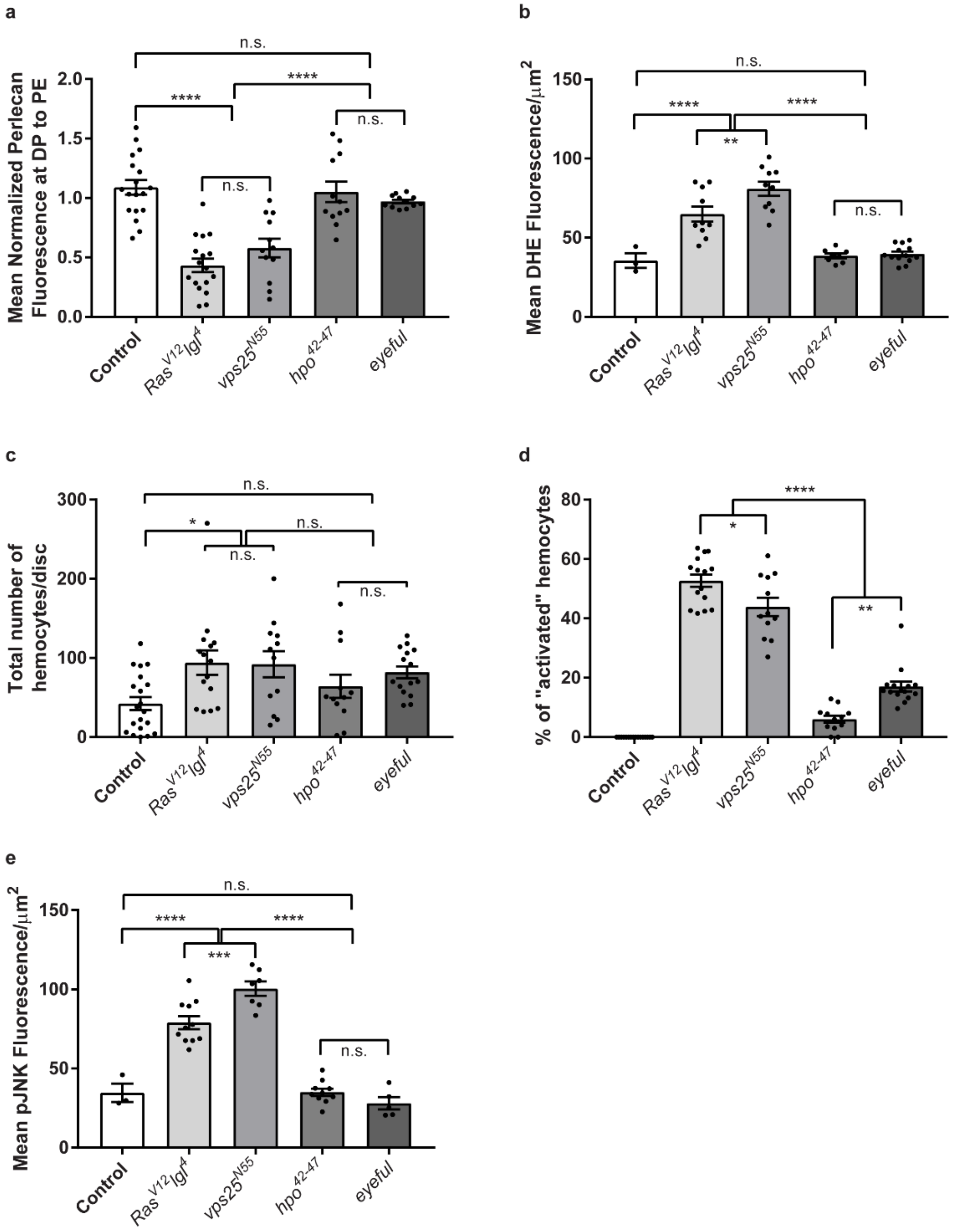

Supplementary Figure 6. Quantifications for Figure 2 and Supplementary Figure 5

(**a**) Quantification of Perlecan intensity in Figure 2 (a, c, e, g) taken from single slices at the basal surface of DP and PE, and represented as a ratio of fluorescence intensity at DP to PE compared to control (*ey-Gal4*) Perlecan intensity ratio. Fluorescence intensity at PE was used as internal control for normalization. Data represented as Mean Fluorescence ± SEM analyzed by one-way ANOVA with Holm-Sidak test for multiple comparisons. ****$p < 0.0001$, n.s. = no statistical

significance. Data from n=18 (control - *ey-Gal4*), 17 (*Ras*^{V12}*lgl*^{4}), 12 (*vps25*^{N55}), 12 (*hpo*$^{42\text{-}47}$) and 12 (*eyeful*) discs analyzed from 3 independent experiments.

(**b**) Quantification of DHE intensity in Figure 2 (b, d, f, h) taken from maximum intensity projections and compared to control (*ey-Gal4*), represented as Mean Fluorescence$\pm$ SEM analyzed by one-way ANOVA with Holm-Sidak test for multiple comparisons. **p=0.0075, ****p < 0.0001, n.s. = no statistical significance. Data from n=3 (control - *ey-Gal4*), n=10 (*Ras*^{V12}*lgl*^{4}), 10 (*vps25*^{N55}), 8 (*hpo*$^{42\text{-}47}$) and 13 (*eyeful*) discs analyzed from 3 independent experiments.

(**c-d**) Quantification of hemocyte recruitment (c) and activation (d) in Supplementary Figure 5 (a, c, e, g), compared to control (*ey-Gal4* from Supplementary Figure 1d, e). Hemocyte activation was measured by the percentage of total hemocytes that had one or more cellular protrusions. Total number and percent activated hemocytes were analyzed by one-way ANOVA with Tukey's multiple comparisons test. *p=0.0168 (c), *p=0.007 (d), **p=0.004, ****p < 0.0001, n.s. = no statistical significance. Data from n=20 (control - *ey-Gal4*), n=15 (*Ras*^{V12}*lgl*^{4}), 12 (*vps25*^{N55}), 12 (*hpo*$^{42\text{-}47}$) and 15 (*eyeful*) discs analyzed from 3 independent experiments.

(**e**) Quantification of pJNK intensity in Supplementary Figure 5 (b, d, f, h) taken from maximum intensity projections and compared to control (*ey-Gal4*), represented as Mean Fluorescence$\pm$ SEM analyzed by one-way ANOVA with Holm-Sidak test for multiple comparisons. ***p=0.0012, ****p<0.0001, n.s. = no statistical significance. Data from n=3 (control - *ey-Gal4*), n=11 (*Ras*^{V12}*lgl*^{4}), 7 (*vps25*^{N55}), 10 (*hpo*$^{42\text{-}47}$) and 5 (*eyeful*) discs analyzed from 3 independent experiments.

Source data are provided as a Source Data file.

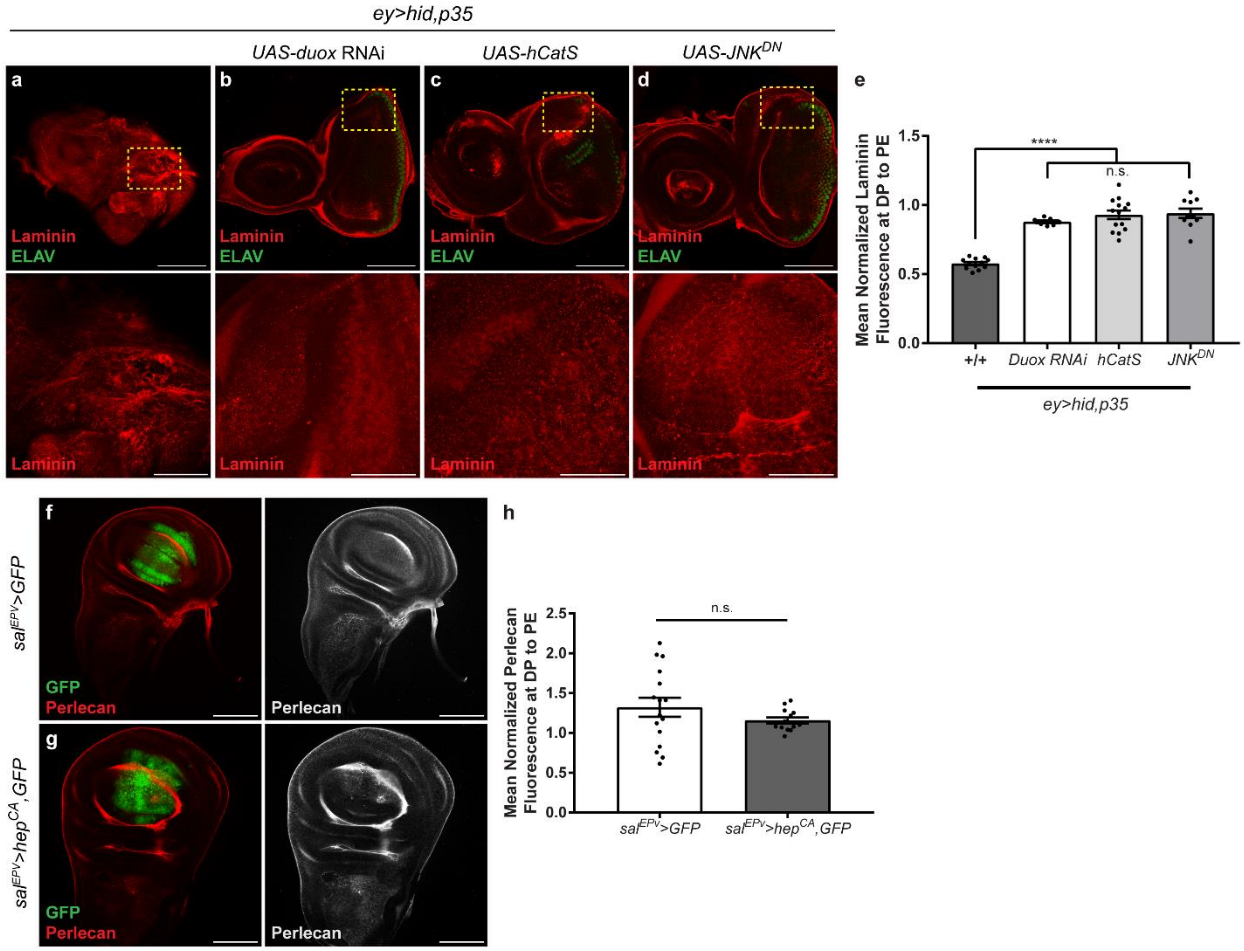

Supplementary Figure 7. Effect of loss of ROS and JNK on Laminin and JNK hyper-activation on BM

(**a-d**) Representative examples of *ey>hid,p35* discs expressing *Duox* RNAi (b), the catalase *hCatS* (c) and JNK^{DN} (d) labeled for Laminin (red) and ELAV (green) compared to *ey>hid,p35*-only (a). Single slices focusing on the basal side of DP (top), yellow squares magnified (bottom), show intact and uniform Laminin labeling upon loss of ROS or JNK. Quantified in (e). Scale bars, 100µm (top); 50µm (bottom)

(**e**) Quantification of Laminin intensity in (a-d) taken from single slices at the basal surface of DP and PE. Fluorescence intensity at PE was used as internal control for normalization. Data represented as Mean Fluorescence ± SEM analyzed by one-way ANOVA with Holm-Sidak test for multiple comparisons. ****p < 0.0001, n.s. = no statistical significance. n=12 (*ey>hid,p35*), 7 (*duox* RNAi), 14 (*hCatS*) and 10 (JNK^{DN}) discs analyzed from 3 independent experiments.

(**f-g**) Hyper-activation of JNK by over-expressing a constitutively activated construct of the JNKK Hep (*hep*^{CA}) in the GFP positive area (g) of the wing pouch does not damage the BM similar to control (f). Perlecan (red left; grey right) is still intact and uniform indicating that JNK activity is not sufficient to damage BM. Quantified in (h). Scale bars, 100μm

(**h**) Quantification of Perlecan intensity in (f-g) taken from single slices at the basal surface of DP and PE. Fluorescence intensity at PE was used as internal control for normalization. Data represented as Mean Fluorescence $\pm$ SEM analyzed by one-way ANOVA with Holm-Sidak test for multiple comparisons. n.s. = no statistical significance. n=16 (*sal*^{EPv}>*GFP*), and 13 (*sal*^{EPv}>*hep*^{CA}, *GFP*) discs analyzed from 3 independent experiments.

Source data are provided as a Source Data file.

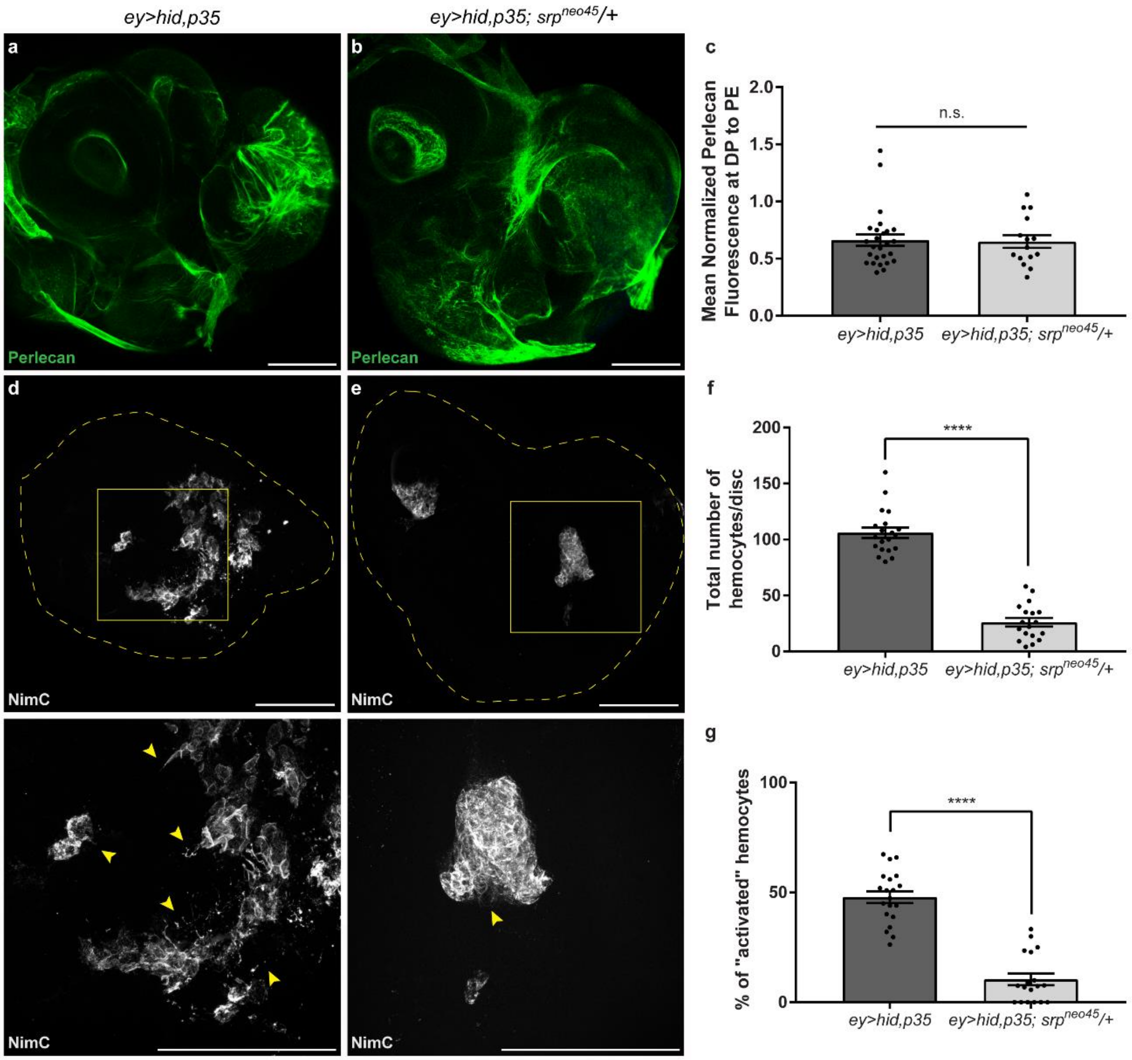

Supplementary Figure 8. Hemocytes numbers do not correlate with BM damage of undead discs

(**a-b**) In *ey>hid,p35* eye imaginal discs, heterozygous *srp*^{neo45} suppresses the overgrowth phenotype, however, the BM is still damaged. Perlecan labeling shows areas of gaps and discontinuities around the discs (a-b, quantified in c). Scale bars, 100μm.

(**c**) Quantification of Perlecan intensity in (a-b) taken from single slices at the basal surface of DP and PE. Fluorescence intensity at PE was used as internal control for normalization. Data represented as Mean Fluorescence ± SEM analyzed by Two-tailed Unpaired T test. n.s. = no statistical significance. Data from n=26 (*ey>hid,p35*), 15 (*ey>hid,p35;srp*^{neo45}/+) discs analyzed from 3 independent experiments.

(**d-e**) Hemocytes detected by NimC (grey; yellow squares magnified bottom). Heterozygous *srp*^{neo45} in *ey>hid,p35* undead discs causes the hemocytes to adopt an inactive morphology, where they form cellular aggregates along the morphogenetic furrow and do not extend cellular protrusions, unlike *ey>hid,35* alone where hemocytes have activated morphology, indicated by yellow arrowheads (bottom). Scale bars, 100μm.

(**f-g**) Quantification of hemocyte recruitment (f) and activation (g) in *ey>hid,p35* and *ey>hid,p35;srp*^{neo45}*/+* discs. Hemocyte activation was measured by the percentage of total hemocytes that had one or more cellular protrusions. Total number and percent activated hemocytes were analyzed by Two-tailed Unpaired T test. ****p < 0.0001. Data from n=20 (*ey>hid,p35*), 18 (*srp*^{neo45}*/+*) discs analyzed from 3 independent experiments.

Source data are provided as a Source Data file.

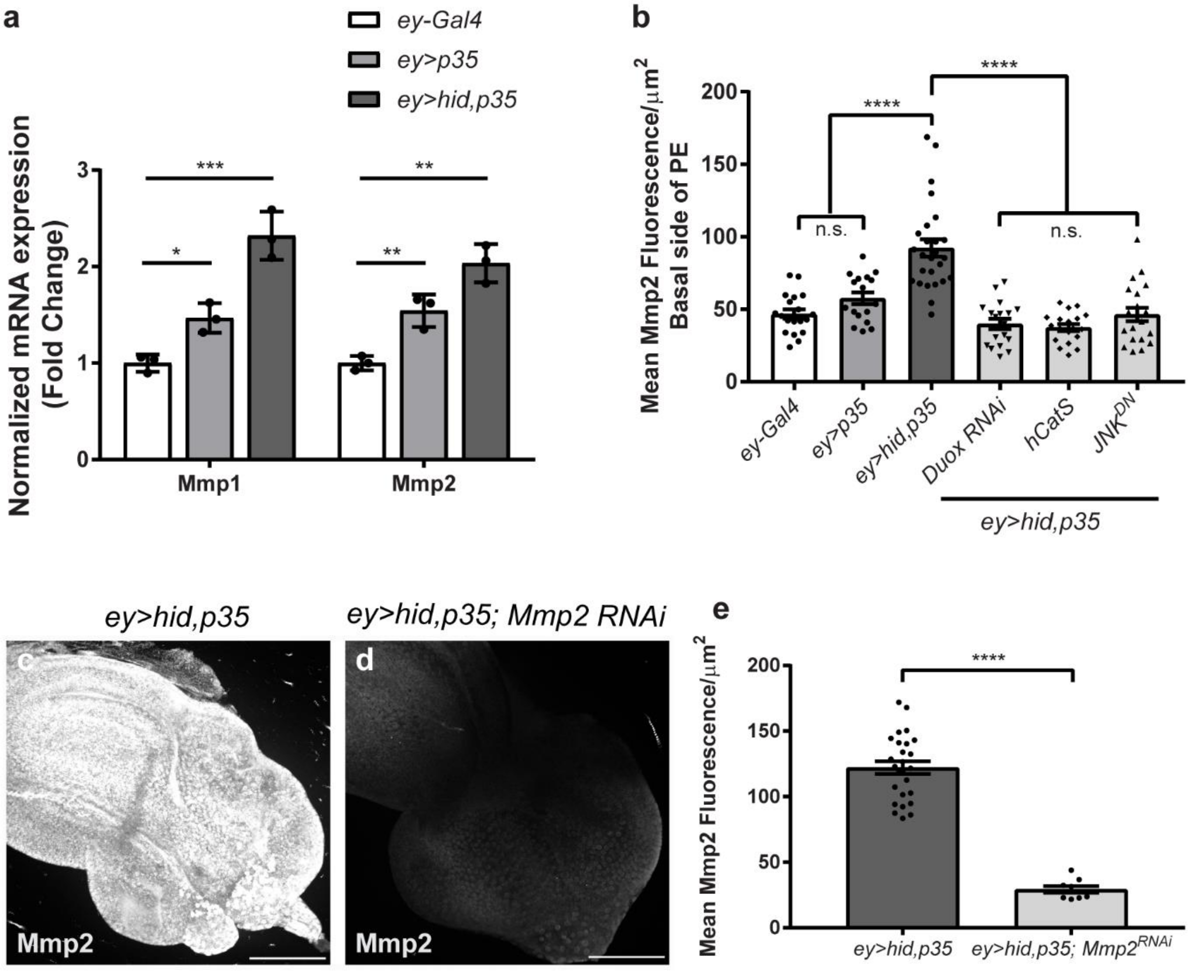

Supplementary Figure 9. MMPs in undead discs.

(**a**) *Mmp1* and *Mmp2* mRNA measured by qPCR. Compared to *ey-Gal4* control eye discs, the relative levels of *Mmp1* and *Mmp2* transcripts are significantly upregulated in undead *ey>hid,p35* eye discs. Graph plotted as mean $\pm$ SD of 3 independent biological replicates and normalized to *ey-Gal4*. p-value calculated by Two-tailed Unpaired T test. P-values *Mmp1*: *p=0.010, ***p=0.0010; *Mmp2*: **p=0.0070 (*ey-Gal4* and *ey>p35*), **p=0.0011 (*ey-Gal4* and *ey>hid,p35*).

(**b**) Quantification of Mmp2 intensity in Figure 4 (a-c; e-g) taken from single slices at the basal surface of the PE. Data represented as Mean Fluorescence $\pm$ SEM analyzed by one-way ANOVA with Holm-Sidak test for multiple comparisons. ****p < 0.0001, n.s. = no statistical significance. Data from n=19 (*ey-Gal4*), 17 (*ey>p35*) and 26 (*ey>hid,p35*), 18 (*duox* RNAi), 19 (*hCatS*) and 20 (*JNK^{DN}*) discs analyzed from 3 independent experiments.

(**c-d**) RNAi against *Mmp2* is efficient and shows almost complete reduction of Mmp2 protein levels in *ey>hid,p35* discs. Quantified in (e). Scale bars, 100µm.

(**e**) Quantification of Mmp2 intensity in (c, d) taken from maximum intensity projections, represented as Mean Fluorescence $\pm$ SEM analyzed by Two-tailed Unpaired T test. ****$p<0.0001$. $n=26$ (*ey>hid,p35*), 9 (*Mmp2* RNAi) discs of analyzed from 3 independent experiments.

Source data are provided as a Source Data file.

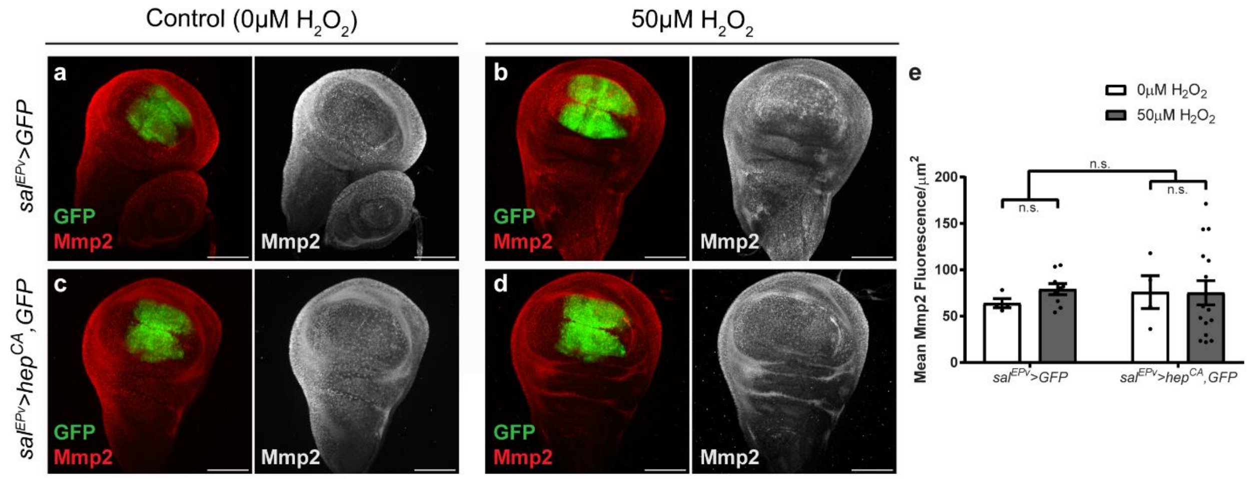

Supplementary Figure 10. ROS and JNK together are not sufficient to induce Mmp2 expression

(**a-d**) Representative examples of wing imaginal discs with wild-type (a-b) or hyper-activated JNK (*hep*^{CA}) (c-d) incubated *ex vivo* in Schneider's media with 0μM H_2O_2 (a, c) or 50μM H_2O_2 (b, d) and labelled with antibody against Mmp2 (red left; grey right). Scale bars, 100μm.

(**e**) Quantification of Mmp2 intensity in *sal*^{EPv} domain (a-d) taken from maximum intensity projections. Data represented as Mean Fluorescence ± SEM analyzed by two-way ANOVA with Tukey's multiple comparisons test. n.s. = no statistical significance. Data from n=4 (*sal*^{EPv}*>GFP* 0μM H_2O_2), 9 (*sal*^{EPv}*>GFP* 50μM H_2O_2), 4 (*sal*^{EPv}*>hep*^{CA}*,GFP* 0μM H_2O_2) and 15 (*sal*^{EPv}*>hep*^{CA}*,GFP* 50μM H_2O_2) discs analyzed from 3 independent experiments.

Source data are provided as a Source Data file.

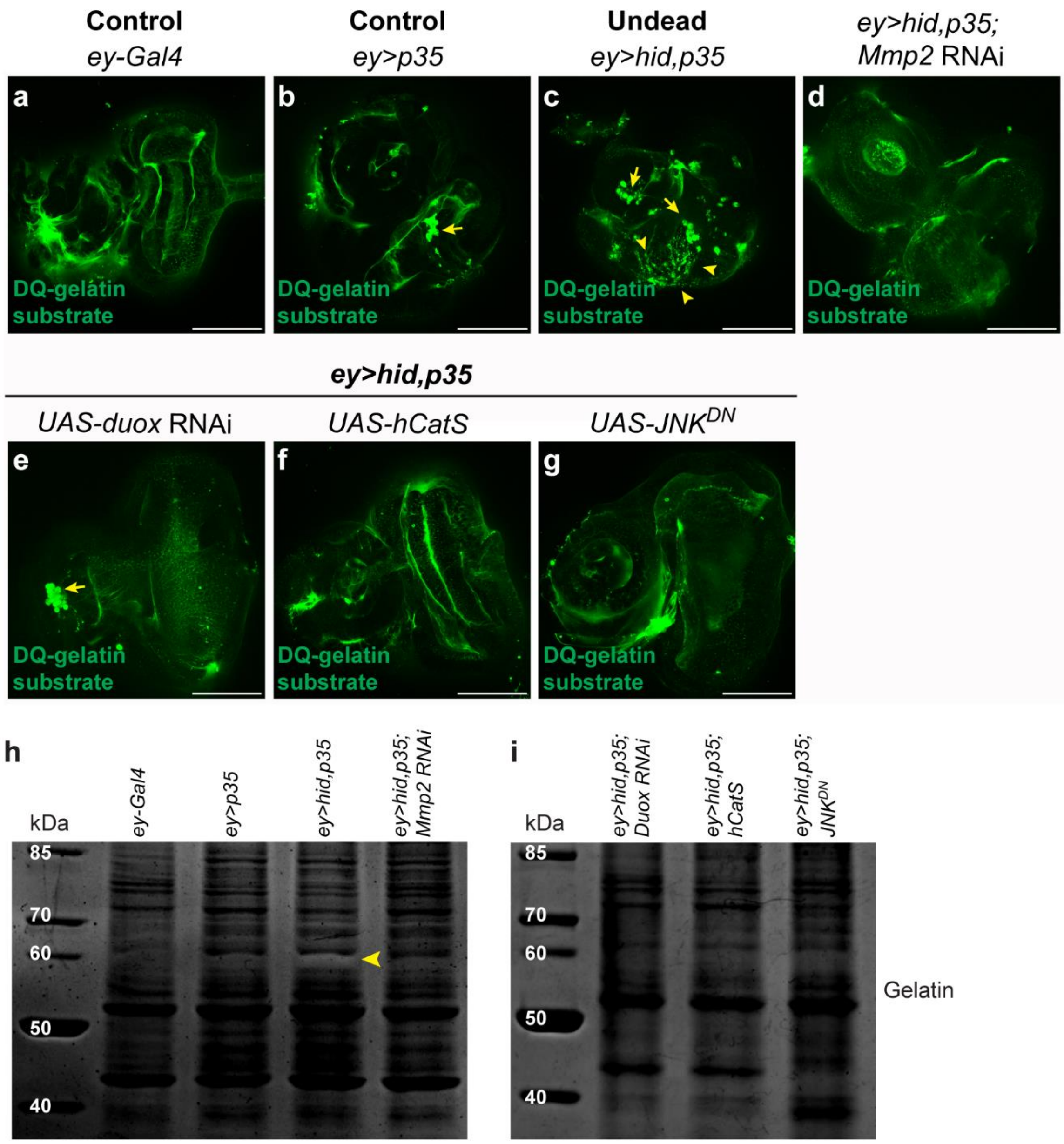

Supplementary Figure 11. Increased Mmp2 activity in undead discs depends on ROS and active JNK

(**a-g**) Representative examples from 3 independent experiments of *in situ* zymography using DQ-gelatin substrate to detect Mmp2 enzymatic activity in *ey>hid,p35* (c) compared to controls (a, b). *ey>hid,p35* discs (c) show an increase of enzymatic cleavage activity towards the fluorogenic DQ-gelatin substrate (yellow arrowheads) visible by small puncta, which are not present in control discs (a, b). Knockdown of *Mmp2* removes these puncta (d) suggesting that Mmp2 accounts for

the enzymatic activity. Similarly, Mmp2 activity is suppressed upon loss of ROS by knockdown of *Duox* (e) or transgenic over-expression of *hCatS* (f), and inactivation of JNK (JNK^{DN}) (g). Hemocytes also label strongly for the fluorogenic substrate (yellow arrows in b, c, e). Scale bars, 100μm.

(**h-i**) Representative gels of gelatin gel zymogram to detect Mmp2 activity. In-gel cleavage of gelatin substrate by Mmp2 causes a clear band on a dark background indicating active enzyme. The protein lysates from *ey>hid,p35* discs have an increased Mmp2 activity as seen by a clear band (yellow arrowhead) that is absent in the controls (h). Knockdown of *Mmp2* suppresses the cleavage of gelatin substrate (h). Loss of ROS by knockdown of *Duox* or transgenic over-expression of *hCatS*, and inactivation of JNK (JNK^{DN}) also suppresses the cleavage of in-gel gelatin substrate (i) indicating reduced Mmp2 activity. Protein molecular weight ladder in kiloDalton (kDa).

Source data containing uncropped gel images are provided as a Source Data file.

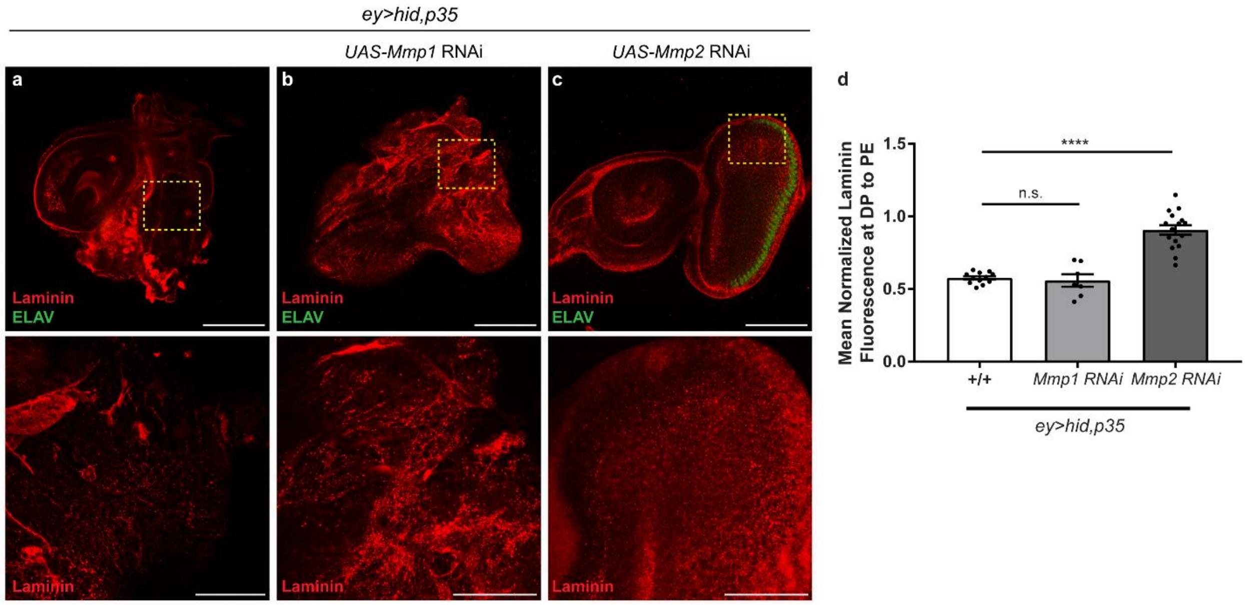

Supplementary Figure 12. Effect of MMP RNAi on Laminin

(**a-c**) Representative examples *ey>hid,p35* discs expressing *Mmp1* RNAi (b) and *Mmp2* RNAi (c) labeled for Laminin (red) and ELAV (green) antibody. Single slices focusing on the basal side of DP (top; yellow squares magnified bottom) show intact and uniform Laminin labeling upon knockdown of *Mmp2*, but no effect on BM damage upon *Mmp1* RNAi compared to *ey>hid,p35*. Scale bars, 100µm (top), 50µm (bottom)

(**d**) Quantification of Laminin intensity in (a-c) taken from single slices at the basal surface of DP and PE. Fluorescence intensity at PE was used as internal control for normalization. Data represented as Mean Fluorescence ± SEM analyzed by one-way ANOVA with Holm-Sidak test for multiple comparisons. ****$p < 0.0001$, n.s. = no statistical significance. n=12 (*ey>hid,p35*), 7 (*Mmp1* RNAi) and 16 (*Mmp2* RNAi) discs analyzed from 3 independent experiments.

Source data are provided as a Source Data file.

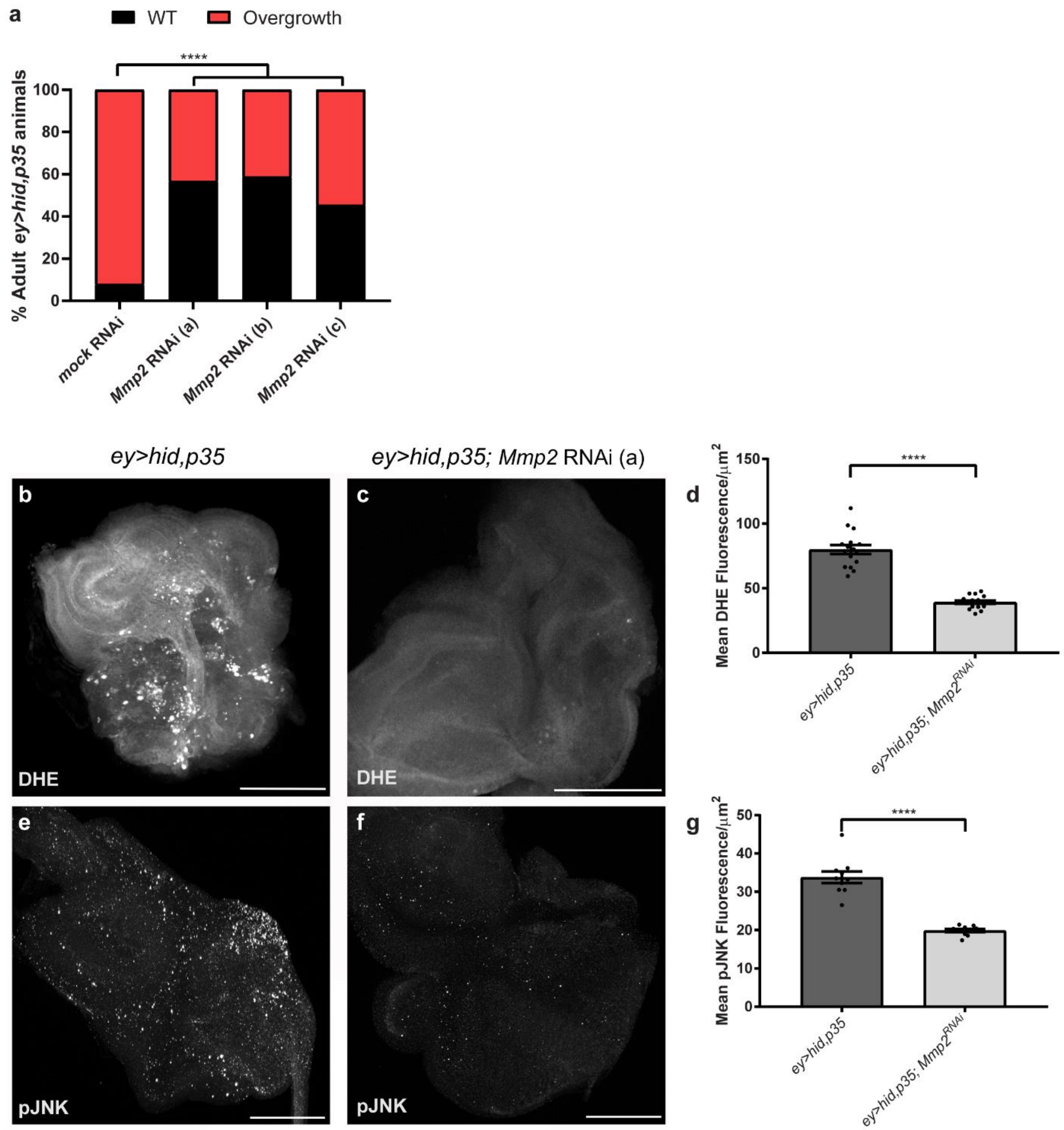

Supplementary Figure 13. Mmp2 participates in the feedback amplification loop of AiP

(**a**) Schematic representation of the effects of three independent *Mmp2* RNAi lines on the *ey>hid,p35* overgrowth phenotype. Based on qualitative screening criteria, progeny are scored as wild-type (WT) (black bars) or overgrown (red bars). Suppression is measured by a shift in percentage to WT from overgrown animals that is significantly different as determined by Two-

sided Fisher's exact test from 3 independent experiments. ****$p < 0.0001$. (n=100 to 150 flies counted per genotype).

(**b-c**) Knockdown of *Mmp2* (c) suppresses ectopic production of ROS (detected by DHE indicator dye) compared to *ey>hid,p35* undead discs (b). Scale bars, 100μm.

(**d**) Quantification of DHE intensity in (b-c) taken from maximum intensity projections, represented as Mean Fluorescence $\pm$ SEM analyzed by Two-sided Unpaired T test. ****$p < 0.0001$. Data from n=16 discs per genotype analyzed from 4 independent experiments.

(**e-f**) *Mmp2* RNAi suppresses activation of JNK (f) observed in *ey>hid,p35* discs (e). Activated JNK is detected by pJNK antibody. Scale bars, 100μm

(**g**) Quantification of pJNK intensity in (e-f) taken from maximum intensity projections, represented as Mean Fluorescence $\pm$ SEM analyzed by Two-tailed Unpaired T test. ****$p < 0.0001$. Data from n=10 discs per genotype analyzed from 3 independent experiments.

Source data are provided as a Source Data file.

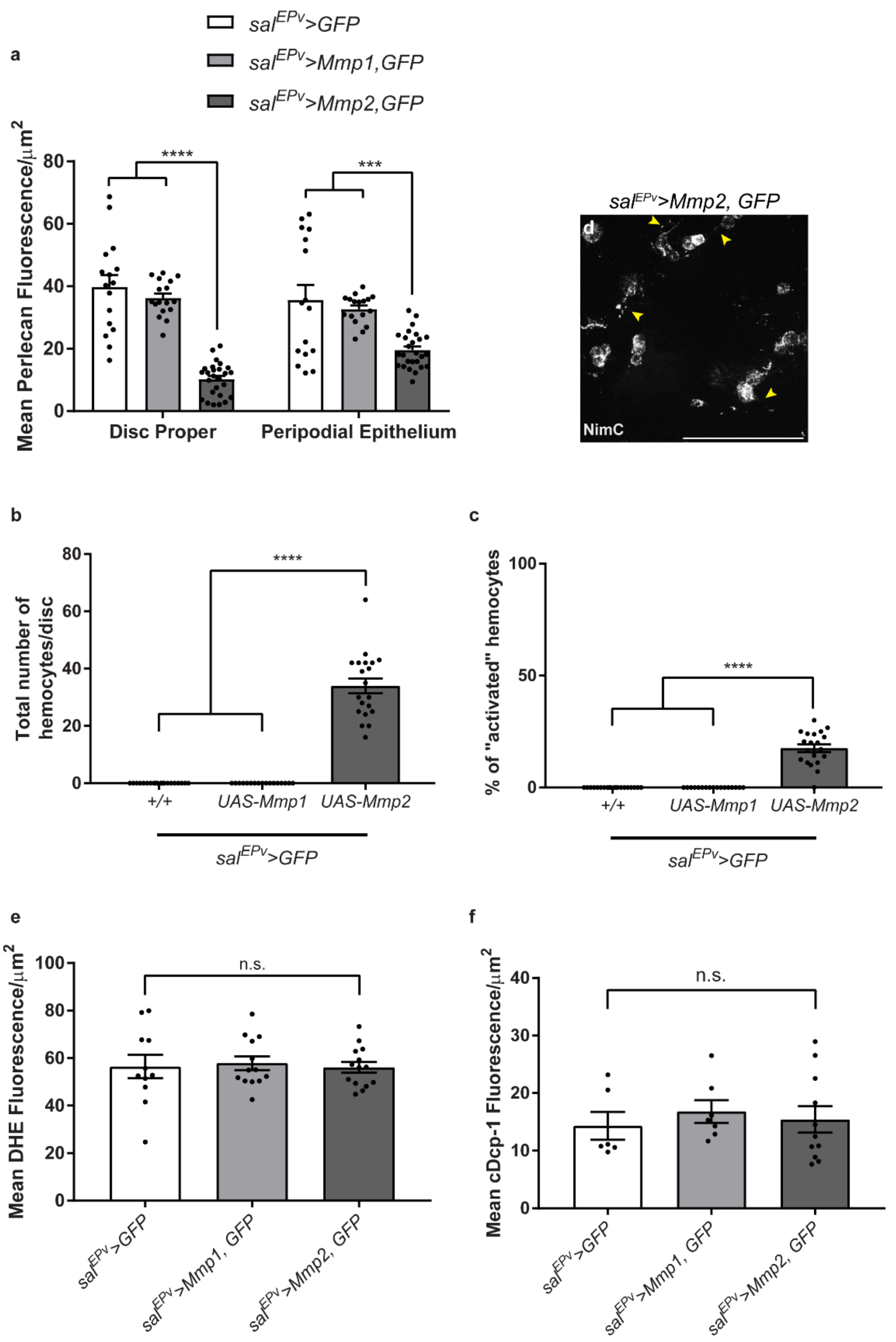

Supplementary Figure 14. Quantifications for Figure 6

(**a**) Quantification of Perlecan intensity in Figure 6a-c taken from single slices at the basal surface of DP and PE. Fluorescence intensity at PE was not used for normalization as PE also showed

damage to BM upon *Mmp2* expression. Data represented as Mean Fluorescence ± SEM analyzed by one-way ANOVA with Holm-Sidak test for multiple comparisons. ***p=0.0002, ****p < 0.0001. Data from n=16 (*sal^{EPv}>GFP*), 17 (*sal^{EPv}>Mmp1, GFP*) and 26 (*sal^{EPv}>Mmp2, GFP*) discs analyzed from 4 independent experiments.

(**b**-**c**) Quantification of hemocyte recruitment (b) and activation (c) in Figure 6d-f. Hemocyte activation was measured by the percentage of total hemocytes that had one or more cellular protrusions. Total number and percent activated hemocytes were analyzed by one-way ANOVA with Tukey's multiple comparisons test. ****p < 0.0001. Data from n=20 (*sal^{EPv}>GFP*), 17 (*sal^{EPv}>Mmp1, GFP*) and 20 (*sal^{EPv}>Mmp2, GFP*) discs analyzed from 3 independent experiments.

(**d**) Representative image of activated hemocytes on *sal^{EPv}>Mmp2, GFP* disc labeled with anti-NimC antibody. Hemocytes with cellular protrusions indicated by yellow arrowheads mark the activated phenotype of hemocytes. Scale bar, 100μm.

(**e**) Quantification of DHE intensity in Figure 6g-i taken from maximum intensity projections, represented as Mean Fluorescence ± SEM analyzed by one-way ANOVA with Holm-Sidak test for multiple comparisons. n.s. = no statistical significance. Data from n=11 (*sal^{EPv}>GFP*), 13 (*sal^{EPv}>Mmp1, GFP*) and 14 (*sal^{EPv}>Mmp2, GFP*) discs analyzed from 3 independent experiments.

(**f**) Quantification of cDcp-1 intensity in Figure 6j-l taken from maximum intensity projections, represented as Mean Fluorescence ± SEM analyzed by one-way ANOVA with Holm-Sidak test for multiple comparisons. n.s. = no statistical significance. Data from n=6 (*sal^{EPv}>GFP*), 7 (*sal^{EPv}>Mmp1, GFP*) and 11 (*sal^{EPv}>Mmp2, GFP*) discs analyzed from 3 independent experiments.

Source data are provided as a Source Data file.
