# Supplementary material for: Basement membrane damage by ROS- and JNK-mediated Mmp2 activation drives macrophage recruitment to overgrown tissue
Source: Nat Commun. 2020 Jul 20;11:3631. doi: 10.1038/s41467-020-17399-8 (PMC7371875; doi:10.1038/s41467-020-17399-8)
Supplement: Supplementary file 3 — Reporting Summary [file 41467_2020_17399_MOESM3_ESM.pdf]

nature research

Corresponding author(s): Andreas Bergmann

Last updated by author(s): Jun 4, 2020

Reporting Summary

Nature Research wishes to improve the reproducibility of the work that we publish. This form provides structure for consistency and transparency in reporting. For further information on Nature Research policies, see our Editorial Policies and the Editorial Policy Checklist.

Statistics

For all statistical analyses, confirm that the following items are present in the figure legend, table legend, main text, or Methods section.

n/a	Confirmed	
☐	☒	The exact sample size (*n*) for each experimental group/condition, given as a discrete number and unit of measurement
☐	☒	A statement on whether measurements were taken from distinct samples or whether the same sample was measured repeatedly
☐	☒	The statistical test(s) used AND whether they are one- or two-sided<br>*Only common tests should be described solely by name; describe more complex techniques in the Methods section.*
☒	☐	A description of all covariates tested
☒	☐	A description of any assumptions or corrections, such as tests of normality and adjustment for multiple comparisons
☐	☒	A full description of the statistical parameters including central tendency (e.g. means) or other basic estimates (e.g. regression coefficient) AND variation (e.g. standard deviation) or associated estimates of uncertainty (e.g. confidence intervals)
☐	☒	For null hypothesis testing, the test statistic (e.g. *F*, *t*, *r*) with confidence intervals, effect sizes, degrees of freedom and *P* value noted<br>*Give P values as exact values whenever suitable.*
☒	☐	For Bayesian analysis, information on the choice of priors and Markov chain Monte Carlo settings
☒	☐	For hierarchical and complex designs, identification of the appropriate level for tests and full reporting of outcomes
☒	☐	Estimates of effect sizes (e.g. Cohen's *d*, Pearson's *r*), indicating how they were calculated

Our web collection on statistics for biologists contains articles on many of the points above.

Software and code

Policy information about availability of computer code

Data collection	Zen Blue and Zen Black (Carl Zeiss) were used for data collection via confocal imaging and quantification.
Data analysis	Image J and Photoshop were used for data analysis; GraphPad Prism was used for statistical analysis and data plotting.

For manuscripts utilizing custom algorithms or software that are central to the research but not yet described in published literature, software must be made available to editors and reviewers. We strongly encourage code deposition in a community repository (e.g. GitHub). See the Nature Research guidelines for submitting code & software for further information.

Data

Policy information about availability of data

All manuscripts must include a data availability statement. This statement should provide the following information, where applicable:

- Accession codes, unique identifiers, or web links for publicly available datasets
- A list of figures that have associated raw data
- A description of any restrictions on data availability

No large data sets were generated in the course of this work. All data are available in the provided figures, supplementary figures and Source data file. Source data underlying Figs. 1, 3, 4, 5 and Supplementary Figs. 1 – 14 are provided as the Source data file.

Field-specific reporting

Life sciences study design

All studies must disclose on these points even when the disclosure is negative.

Sample size	No sample size calculation was performed. Sample size was chosen based on previously published reports of similar nature.
Data exclusions	The obtained data were very similar and there was no data exclusions.
Replication	Experiments were replicated at least three independent times to ensure consistency and reproducibility of data.
Randomization	Samples were allocated into experimental groups based on genotype.
Blinding	Blinding was not necessary because there was no experimental bias.

Reporting for specific materials, systems and methods

We require information from authors about some types of materials, experimental systems and methods used in many studies. Here, indicate whether each material, system or method listed is relevant to your study. If you are not sure if a list item applies to your research, read the appropriate section before selecting a response.

Materials & experimental systems

n/a	Involved in the study	
☐	☒	Antibodies
☒	☐	Eukaryotic cell lines
☒	☐	Palaeontology and archaeology
☐	☒	Animals and other organisms
☒	☐	Human research participants
☒	☐	Clinical data
☒	☐	Dual use research of concern

Methods

n/a	Involved in the study	
☒	☐	ChIP-seq
☒	☐	Flow cytometry
☒	☐	MRI-based neuroimaging

Antibodies

Antibodies used	Commercially available antibodies: Rabbit anti-Laminin (Abcam #ab47651); Rabbit anti-cleaved Dcp-1 (Cell Signaling Technologies #9578S): Rabbit anti-phospho JNK (Promega #V7931); Rat anti-ELAV (DSHB #7E8A10); Mouse anti-Mmp1 (DSHB #14A3D2). Donations from private investigators: Rabbit anti-Perlecan (kind gift from Stefan Baumgartner); Mouse anti-NimC (P1a,P1b, kind gift from István Andó), Rabbit anti-Mmp2 (#436, kind gift from Kendal Broadie)
Validation	Antibodies were validated by immunoblotting or immunohistochemistry under conditions where the corresponding antigen was not present (mutants) or overexpressed. Only antibodies which passed these tests were used in this study.

Animals and other organisms

Policy information about studies involving animals; ARRIVE guidelines recommended for reporting animal research

Laboratory animals	Drosophila melanogaster was used in this study. 5-6 day old larvae (late 3rd instar) and 3-5 day old adult animals were used. Both males and females were analyzed.
Wild animals	No wild animals were used in this study.
Field-collected samples	The study did not involve field-collected samples.
Ethics oversight	No ethical approval is required for working with Drosophila melanogaster.

Note that full information on the approval of the study protocol must also be provided in the manuscript.
